# Reliability and validity of mental health measurement of young people in a Chinese urban context

**DOI:** 10.1371/journal.pone.0321523

**Published:** 2025-06-17

**Authors:** Jing Zhou, Timo Toikko

**Affiliations:** 1 School of Law, Shanghai Lixin University of Accounting and Finance, Shanghai, China; 2 Department of Social Sciences, University of Eastern Finland, Kuopio, Finland; Kazimierz Wielki University in Bydgoszcz: Uniwersytet Kazimierza Wielkiego w Bydgoszczy, POLAND

## Abstract

Globally, there is increasing attention on mental health promotion for young people because of its great public health and social significance. The scarcity of suitable mental health measures for young people underscores the urgent need for tailored interventions to address their unique mental health challenges. This study explores the reliability and validity of the self-rated mental health measurement scale (SRMHS) for assessing Chinese youth mental health in an urban context in China, addressing a critical research gap in culturally and contextually appropriate mental health measurement instruments. The study responds to the global demand for valid and reliable health data to support mental well-being initiatives. Utilizing a sample of 3,279 participants aged 14–35 years, the study employed rigorous statistical analyses, including Exploratory Factor Analysis (EFA), Confirmatory Factor Analysis (CFA), and tests for convergent, discriminant, and concurrent validity, to evaluate the scale’s psychometric properties. Results confirm a three-dimensional structure of the mental health scale, encompassing positive emotion, psychosocial symptoms and negative emotion, and cognitive function, demonstrating good internal consistency, reliability, and validity. Subgroup consistency categorized across gender, age, and social identity was examined, and the significant correlations between mental health scores with relevant psychological outcomes showed good concurrent validity, indicating the effectiveness of SRMHS in capturing a broad spectrum of psychological states relevant to youth development. Findings highlight the importance of a multidimensional approach to mental health assessment, reflecting global advocacy for recognizing mental well-being as not merely the absence of illness, but also the presence of positive psychological states. This study significantly contributes to the field by providing a scientifically robust and culturally sensitive tool for assessing youth mental health in China, and promotes a more inclusive and holistic approach to mental health research and practice globally. The results of this study could benefit policy-making, targeted interventions, and the overall improvement of youth mental well-being.

## 1. Introduction

### 1.1. Youth mental health in the global context

There is a growing focus on assessing well-being and mental health across various settings and services beyond traditional mental health care environments, particularly for adolescents and young adults [1–3]. Adolescence and young adulthood are crucial periods marked by significant biological, psychological, and social transitions which may influence lifelong health and wellbeing. Individuals experience the pivotal stage in the development of physical, mental, and social health, serving as a foundation for health trajectories throughout their life course [4,5]. The mental health of young people is a matter of significant concern due to its profound implications for individual well-being and socioeconomic outcomes, including impaired functioning, increased risk of mental disorder, substantial healthcare expenditures, and a greater burden on social services [6–8], which could cause further reductions in the investment of human capital assets, such as education and health [9,10]. Nonetheless, adolescents and young adults have been overlooked generally in public initiatives because they are generally considered to be the healthiest group in the society [10,11]. Globally, the growth of the youth population and the broad perspective of prioritized youth development have drawn attention to their diverse health needs. In recent years, the discussion on youth mental health is taking place online increasingly via social media platforms. In 2020, the PMNCH (Partnership for Maternal, Newborn and Child Health) of the World Health Organization (WHO), in partnership with other youth organizations, developed a consensus framework for defining, programming, and measuring youth health, and discussed the relationship between youth health and their well-being [12]. The comprehensive framework also included a multistakeholder Call to Action to prioritize youth health [13]. A growing number of countries have significantly improved their investment in youth mental health care services in order to alleviate the considerable disease burden stemming from mental health issues for these age groups [14,15]. The phrase “youth are the future” has become a universal consensus of global political entities [16], and the health well-being of the youth has been recognized by the United Nations as the third goal of “the 2030 Agenda for Sustainable Development” [17].

However, adolescents and young adults refers to a diverse group of young people spanning different age ranges, involving significant developmental transitions from early adolescence to young adulthood [3,18]. They face varied psychological challenges as they navigate key developmental stages. In-school adolescents experience intense pressure from academic performance, especially in extremely demanding and competitive education system like China’s *gaokao*, leading to heightened stress, anxiety, and emotional instability [19]. Social comparison through social media exacerbates feelings of inadequacy, while peer pressure influences risk-taking behaviors [20]. Adolescents also face identity formation challenges, particularly around sexual and gender identity, which can be stressful, especially in unsupportive environments [21]. For working young adults, career uncertainties, financial pressures, and societal expectations around securing stable jobs and forming relationships contribute to significant stress. The transition from school to work brings added responsibilities, with many facing burnout from long hours and societal pressure to meet life milestones [22]. Isolation and disconnection from social support networks further compound these challenges, often manifesting as anxiety or depression [23]. Unemployed or out-of-school youth, particularly in contexts where academic and professional success is highly valued, struggle with feelings of failure, social isolation, and stigma [24]. The lack of clear direction and limited support resources can lead to a sense of hopelessness, making them more vulnerable to mental health issues and risky behaviors. Such pressures in Chinese context are amplified by the cultural and societal environment. Adolescents are under immense pressure to excel academically, with family honor often tied to their success, resulting in stress and anxiety around exams like the *gaokao* [25]. Young adults face societal expectations to quickly secure stable employment, housing, and marriage, contributing to financial and emotional strain [26]. The rapid pace of urbanization and cultural shifts, combined with traditional values, often leads to identity conflicts [27]. Additionally, mental health stigma remains a barrier, preventing many from seeking help, further intensifying their psychological burden [28].

Global health reports have highlighted an ongoing demand for valid and reliable health data, especially since mental health issues have emerged as one of the most significant disease burdens among adolescents [6,10]. As the current emphasis is on promoting youth mental wellbeing, practitioners require accessible, reliable, and valid measures to assess improvements in psychological wellbeing effectively, avoiding the limitations of “ceiling effects” found in traditional scales focused on mental illness or poor wellbeing [29]. The previous theoretical and empirical literature has addressed and commonly agreed on the definition of mental health, which is not merely the absence of mental illness, but also involves presenting positive psychological status [30–32]. However, research on comprehensive and generic mental health among young people, not just from a deficit perspective, is still underdeveloped [6,11,29,33–35]. Additionally, general mental health measurements offer distinct advantages by being generic in nature, which reduce the necessity for multiple, diagnosis-specific tests that are often pragmatically challenging to administer [36], facilitate comparisons between different cases and services [37], and benefit the process of policy-program planning, monitoring, and adjusting [38]. Distinguished from professional structured diagnostic instruments of mental disorder, brief mental health scales are recognized as a much more useful and efficient screening tool to evaluate the mental health status of individuals [39]. The core issue is the scale’s validity as a measurement, which presents the discrimination ability among individuals.

### 1.2. Mental health assessment instruments

Most of the measures in existing limited studies have been found only within adolescents, but there is a lack of accessibility to younger adults. Young people at the earlier stage of adulthood seem to be a missing population in universal health coverage [40]. The youth period is a critical and extended stage of life, including early adolescents, late adolescents, and young adults. Throughout this unique period, individuals undergo special developmental transitions with rapid changes, such as further education, employment, marriage, and childbirth, making them particularly vulnerable to psychological challenges at different age phases. This suggests that it is necessary to operate age-disaggregated studies for young people, making the evidence more precise and targeted for service program planning. Therefore, the instruments measuring youth general mental health are needed to evoke more studies on mental well-being among young people, and to develop practical interventions to promote well-being and prevent mental health problems [34].

According to the previous literature, while there is much more attention on youth psychosocial well-being and more relevant studies in high-income countries, little is known about mental health status in other low- or middle-income countries [41]. The lack of validated measurements in these contexts maybe partially contributed to the lack of such knowledge or information [42]. The absence of quality research evidence presents a significant barrier to effective local policy-making and mental healthcare responses. Furthermore, to our knowledge, the majority of existing tools for measuring mental health were designed in the context of developed countries and are predominantly available in English. The relevant studies in non-English speaking countries or regions mainly used the translated versions, which brought a significant challenge for the distinct cultural backgrounds, leading to issues of cultural incompatibility, and also raising the question of whether these adapted measures are merely perpetuating western psychological definitions, rather than offering a truly global perspective on mental health [43,44]. The development and application of mental health measurement tools for young people, especially in non-Western contexts, underscores a critical gap in global mental health research. Although many of the tools have been validated in various cultural settings, this practice raises significant concerns tied to the ongoing debates around decolonizing mental health. Questions about what constitutes knowledge and “evidence” in global mental health, and who has the authority to determine what counts as “evidence” are increasingly relevant [6,45,46].

Cultural norms, values, and expressions of psychological states may vary greatly from one culture to another. Different populations may have a distinct understanding of mental health and unique manifestations of health status. This discrepancy underscores the urgent need for more localized research to develop instruments that are culturally sensitive and contextually relevant. Measurement scales that are culturally adapted to specific cultural, linguistic, and social contexts enhance their validity, and ensure that they accurately capture individuals’ experiences and perceptions of mental health. Tailoring measurements would also ensure a more accurate assessment of mental health, that resonates with the local population’s specific expressions and experiences, thereby enhancing the validity of research findings and the effectiveness of subsequent policy and interventions designed to address mental health issues. Thus, a focus on scale development and validation studies is crucial in mental health science [47]. Such an approach would not only address the unique challenges faced by the youth in both developing and developed countries, but also enrich our understanding of mental health by incorporating diverse experiences and strategies.

Moreover, research has found that the length of the utilized measurements varies conditionally. There are many factors that should be taken into account, including the burden of youth participants, time restraints, fidelity to the factor structure of scale, and so on [29]. With respect to our purpose presented earlier, how a more balanced picture of youth general mental health is described, is critical to the development and adherence to the monitoring evaluation of a program designed to improve youth mental well-being.

### 1.3. Youth mental health studies in the Chinese context

In recent years, China has been undergoing a period of social transformation, characterized by an accelerated pace of life and intensified social competition. It has significantly raised psychological stress levels among Chinese citizens, making mental health issues more pronounced, especially among adolescents and young adults. In response to these challenges, China has placed significant emphasis on youth development, particularly concerning youth mental health, aligning with the strategic visions outlined in key policy documents such as the “Healthy China 2030” [48], and the “Medium- and Long-term Youth Development Plan (2016-2025)” (MLYDP) [49]. These policy initiatives and current action plans underscore the national commitment to fostering a supportive environment and offering various resources for sustainable well-being development among young people. They reflect a growing awareness of the importance of mental health in achieving a healthy and harmonious society, and acknowledge the impact of mental health on educational achievements, social integration, and the overall quality of life [50]. Meanwhile, enormous research on youth development including mental health is evolving, with a focus on addressing the unique needs of the youth population through culturally-sensitive assessments and interventions [51]. Although significant progress has been made, ongoing efforts are required to expand access to mental health services. Some cities stand out as pioneers, excelling in the development of services and research focused on youth growth, on youth mental well-being particularly and currently [52–54]. However, there is no nationally recognized and standardized measurement for mental health. Similar to what has been noted earlier in this paper, there are many instruments measuring Chinese people’s mental status, but most of these are translated versions and they focus on negative aspects [51], while targeting specific groups (children, students, the elderly, patients, etc.). Additionally, Chinese youth are defined as aged 14–35 years in the MLYDP, which ranges over a relatively long period. Thus, it is challenging, but important and meaningful, to facilitate the development of age-appropriated and culturally valid instruments that measure the general mental health of adolescents and young adults in the Chinese context.

Currently, the self-rated health measurement scale (SRHMS) is a widely-used instrument in health assessment in China. The SRHMS was independently developed by Xu Jun and his team [55,56]. This scale emerged from a comprehensive dialogue on the definition of health, incorporating insights from established health measurement scales such as Medical Outcomes Study (MOS), Health Information Exchange (HIE), Nottingham Health Profile (NHP), Social Information Processing (SIP), and Short Form Health Survey with 36 items (SF36). Employing methodologies like the Delphi method and on-site investigations, the research team formulated a set of self-rated questions (48 items) which are categorized into 3 subscales: physical health (18 items), mental health (16 items), and social health (13 items) plus with an overall evaluation of health status. Each dimension includes 3 dimensions and an overall evaluation as well. In other words, SRHMS was structured with three subscales, and either of them could be used as an independent tool to measure the targeted aspect of health status. While the summarized scores (44 items) of the three subscles could be recognized as the general self-rated health status. The 4 overall rated items were used as the concurrent validity. All the items used a 0–10 grade scoring method. Since its development, the SRHMS was applied to evaluate the health status of people aged over 14 years in China, and its reliability and validity were examined across diverse groups [56–60]. Previous studies mainly focused on specific groups, such as medical workers, patients, urban/rural resident’s, the elderly, some on college students to test its psychometric properties, but rarely focus on Chinese youth group. The results of recent validation studies showed a relatively consistency that SRHMS had good reliability and validity, however, the applicability of certain items within these scales varies across different groups [58,61–63], which implied that modification may needed when SRHMS was applied to different population. For example, a study of Chinese medical staff demonstrated its Cronbach’s α with result greater than 0.8, and a good fit of confirmatory factor analysis with χ^2^/df = 3.637, NFI = 0.924, RFI = 0.915, IFI = 0.944, TLI = 0.937, CFI = 0.944, RMSEA = 0.046. Meanwhile, it found that 3 items with negative expression should be deleted because their low discriminability in medical groups. Some other studies also supported such finding [58].

### 1.4. Study aims

Considering the comprehensive background provided earlier in this paper, and based on the solid and extensive study foundation of the SRHMS, the aim of the present research is to validate the applicability of the mental health scale part among Chinese adolescents and young adults. To the best of our knowledge, this is the first time there has been an examination of the SRHMS and its use among the extended youth group. Further, the aim is to ensure it is a reliable and valid tool for capturing the nuanced mental health profiles of young people, thus addressing the need for culturally and contextually appropriate mental health measurements within this age group. Additionally, to our knowledge, the subgroup consistency validation of mental health measurement instruments is also lacking in the body of literature. The stability of the measurements could vary due to the differences in characteristics between subgroups [64]. The social identity status of young people varies a lot, depending on their current life course, which mainly involves being students, employed youth, or unemployed youth. Therefore, it is necessary to test the subgroup consistency validation of mental health instruments to ascertain the model fit in different subgroups.

Taken together, given the challenging circumstance of the Chinese context for youth mental health, the goals of this study are to examine the reliability and validity of self-rated mental health outcome as a useful potential measure of youth general mental well-being, which could fill the research gap between high income countries and low-to-middle income countries, thus responding to the continued need for valid and reliable health data. Specific goals were to (1) evaluate the internal consistency of the mental health scale; (2) validate the internal structure of the dimensions of distinct symptoms; (3) examine the construct validity, including convergent and discriminant validity of the scale; (4) analyze the subgroup consistency and measurement invariance, and (5) examine the concurrent validity through testing the associations of youth mental health with overall self-rated mental health (SRMH), overall self-rated health status (SRH), sense of happiness (SH), and meaning in life (ML), which were found to have significant correlations with individual’s mental health [65–68].

## 2. Materials and methods

### 2.1. Instrument applied in the study

#### 2.1.1. Mental health scale.

According to SRHMS developers, the mental health scale is divided into 3 dimensions, respectively: positive emotion (M1), psychosocial symptoms and negative emotion (M2), and cognitive function (M3). Using the method of 1–5 grade scoring for each item, the score of each dimension (M1, M2 & M3) was the summarized score of all items within the dimension. MH was calculated as the total score of all items. A higher score represents a better mental health status. In the measure, the negative valence measures were interspersed with positive valence measures, which were all psychometrically sound [69]. Items 6–12 are negative phrasing, and need reverse scoring when calculated. The order of the questionnaires in the study by those authors is the same in the present study; as in, starting with demographic data.

#### 2.1.2. Concurrent validity measures.

Although self-rated health questions are simple tools, increasing evidence supports their effectiveness as valid measures of individual health status [70,71], as well as happiness [72]. We uesd overall self-rated health (SRH) by asking “Compared to others in your age group, how would you rate your overall health condition?” Additionally, overall self-rated mental health status (SRMH) was measured through the question, “How would you evaluate your general mental health condition? Based on this approach, we measured respondents’ sense of happiness (SH) by asking, “Do you feel happy now in general?” The responses are on a 5-point scale, with high number indicating better general status.

Many previous studies emphasize the importance of meaningful engagement, particularly for adolescents and young adults, in preventing mental illness and enhancing mental well-being [73,74]. Consequently, we included “meaning in life” (MIL) as a crucial criterion for validity. MIL was assessed using the Chinese version of the Meaning in Life Questionnaire (MLQ) [75], which is the most extensively utilized questionnaire with robust psychometric properties. This scale comprises 10 items, utilizing a seven-point Likert scale ranging from 1 (absolutely disagree) to 7 (totally agree). Higher summed scores on this scale indicate a greater sense of meaning in life. The Cronbach’s alpha coefficient for the total scale was 0.853, indicating high reliability.

### 2.2. Sampling and procedure

Youth is a stage of life which now includes adolescents, teenagers, and young adults and is most often used synonymously with young people [22,75]. For statistical purposes, youth are most often, but not exclusively, defined as individuals aged 15–24 years in the global context. In China, youth are defined as aged 14−35 years in the MLYDP. This study used a multistage stratified sampling method to obtain the sample data in Shanghai, China, which is the first city to implement the policy of a Youth Development Plan. As part of the Youth Development Plan, local officials administered a survey on youth in 2023. The recruitment period of participants was from April 13^th^ to the end of May, and the data entry for the paper questionnaires concluded on June 18^th^. All the participants were informed about the nature of the survey, including the parents if the participants aged 14–17 through their schools. All the participants were oral informed the right to refuse or quit the study when they were invited. The research procedures have been reviewed and approved by the ethics committee of School of Law, Shanghai Lixin University of Accounting and Finance with No. C-22-6701-23− 016, which was conducted in accordance with the guidance of the Helsinki Declaration. Finally.

In total, 4370 participants completed the anonymous paper-and-pencil questionnaires. After data screening to remove logically inconsistent and invalid responses, 3,924 responses were valid. For the present study, only participants who completed the relevant key variables were included in the analyses. Accordingly, 3279 participants with complete answers were retained for analysis; participants with missing data on these variables were excluded.

### 2.3. Statistical analyses

Statistical analyses were conducted using IBM SPSS Statistics for Windows, Version 29 [76], and confirmatory factor analysis was performed using IBM SPSS Amos 26.0 [77].We first used Pearson’s correlation analysis to examine the relationships among the 15 individual items, which assessed the inter-item correlations within the scale to ensure the suitability of the data for factor analysis. Followed by it, we evaluated the normality of the distribution of each item by examining both skewness and kurtosis. Then, we tested reliability by using Cronbach’s alpha coefficient. After it, a split-half reliability methodology was adopted through SPSS, and the total sample was split randomly into two groups of 1637 and 1642, respectively. Group of 1637 is for exploratory factor analysis (EFA), and the other group of 1642 is for confirmatory factory analysis (CFA). EFA was conducted on the data to identify the underlying factor structure of items. Analyses used principal component analysis (PCA) with varimax rotation within SPSS to estimate factor loadings. Then, CFA was conducted to evaluate and refine the resulting scale. Maximum Likelihood (ML) would be a good choice as model estimator in accordance with recommendations for modeling data of this type [78,79].

In this study, several types of validation procedures were conducted to evaluate the psychometric properties of the scale, including construct validity, concurrent validity, and subgroup consistency testing. Specifically, construct validity was evaluated by examining both convergent validity and discriminant validity using average variance extracted (AVE), composite reliability (CR), and the Fornell-Larcker criterion.

Usually, chi-square (χ^2^) or relative chi-square (χ^2^/df) is commonly used as an index in CFA, but it is sensitive to sample size, and would nearly always reject the model when the sample size is large [80–82]. Thus, combined goodness-of fit indices were applied to evaluate the model fit, which included root mean square error of approximation (RMSEA), standardized root mean square residual (SRMR), Tucker-Lewis index (TLI), comparative fit index (CFI), and the accepted values of these indices are displayed below [80]. Models were adjusted in accordance with modification indices, with the requirement that the initial model should fit reasonably well, and the modifications should be justifiable theoretically [81]. Then, subgroup consistency was examined across gender, age, and social identity.

Afterwards, considering there is no gold standard for mental health measurement (to our knowledge), we used overall SRMH, SRH, SH, and ML as a concurrent criterion to validate the mental health scale. Self-reported mental health status is a commonly used subjective assessment method to evaluate individuals’ overall mental health status, which is based on a question, asking individuals to rate their overall health, usually with Likert’s response options (excellent, very good, good, fair, and poor), and so this is overall mental health in this context [65]. Sense of happiness and meaning in life were also included as criteria in the analysis. Pearson‘s r was examined on the mental health total scale (summed) score, as well as the sub-dimensional summed scores, based on the factors derived from CFA.

## 3. Results

### 3.1. Descriptive data

Participants were 14–35 years of age (M ± SD 24.68 ± 5.83) and 46.7% participants were male youth. The age groups were further divided based on typical educational and social role transitions observed among Chinese youth, corresponding to the stages of secondary education (14–18), higher education (19–24), early career entry (25–30), and early mature adulthood (31–35). There are three subgroups regarding their social identity, including students, employed youth, and unemployed youth. Because of age range and policies on marriage and fertility, 78.65% participants were unmarried, and 69.01% were only-child in their family. The sample characteristics are shown in Table 1. We randomly divided the overall population into 2 groups and compared the distribution of these demographic variables in the two groups using the Chi-square Test to determine if there were significant differences. The results indicated that there were no significant differences in the distribution of these variables between the two groups (p > 0.05).

**Table 1:** Demographical characteristics of the participant (n = 3279).

		Total Sample		EFA sample (n = 1642)		CFA sample (n = 1637)	
	Characteristics	Number	Percent (%)	Number	Percent (%)	Number	Percent (%)
Age group	14-18	625	19.06	294	17.90	331	20.22
	19-24	928	28.3	484	29.48	444	27.12
	25-30	1100	33.55	553	33.68	547	33.41
	31-35	626	19.09	311	18.94	315	19.24
Gender	Male	1532	46.72	755	45.98	777	47.46
	Female	1747	53.28	887	54.02	860	52.54
Household individual (Hukou)	Citizenship	2129	64.93	1072	65.29	1057	64.57
	Non-citizenship	1150	35.07	570	34.71	580	35.43
Social identity	students	1077	32.84	529	32.22	548	33.48
	working youth	1782	54.35	901	54.87	881	53.82
	unemployed youth	420	12.81	212	12.91	208	12.71
Only-child status	Yes	2263	69.01	1121	68.27	1142	69.76
	No	1016	30.99	521	31.73	495	30.24
Marital status	Non-married	2579	78.65	1289	78.50	1290	78.80
	Married experience (including current married, divorced, separated, widowed)	700	21.35	353	21.50	347	21.20

### 3.2. Internal structure: Item distributions, inter-item correlations, and reliability

Across the 15 items, shown in Table 2, mean scores ranged between 2.68 and 3.95, within a possible range of 0–5, and the standard deviations ranged from 0.782 to 1.125. Besides, one item for self-rated overall mental health (SRMH) was calculated too, and showed a score of 3.68 with SD 0.885. Previous studies suggest that ordinal data can be reasonably treated as continuous for the purposes of structural equation modeling (SEM) [83]. In our study, we treated the 5-point Likert scale responses as continuous variables, verifying their normal distribution characteristics through skewness and kurtosis analyses of the total sample (see Table 2) and two randomly divided subsamples (see S1 Table in S1 File). The result of independent-samples T test showed that there was no group difference between the subsamples (p > 0.05). Then we computed Cohen’s d values ranging from 0.000 to 0.065, confirming that the differences between the subsamples are minimal [84], which supported the robustness of the data for later EFA and CFA. After that, we conducted Pearson correlation test, and most of the Pearson correlation coefficients were significant (p < 0.01) except for the relations between item 9 and item 14/15, and the value ranged from 0.106 to 0.738 if item 9 was excluded (see S2 Table in S1 File). It indicates that the underlying factors would be moderately correlated, which supports our theoretical expectation for the internal structure analysis of the mental health scale.

**Table 2:** Descriptive statistics for the 15 mental health scale items and SRMH item (n = 3279).

	Mean	SD	Skewness	Kurtosis
MQ1: be optimistic about the future	3.64	0.979	−0.314	−0.392
MQ2: be satisfied with current living conditions	3.57	0.950	−0.243	−0.406
MQ3: have confidence in yourself	3.69	0.955	−0.249	−0.557
MQ4: feel safe in daily living environment	3.95	0.927	−0.586	−0.220
MQ5: feel happy	3.77	0.923	−0.302	−0.472
MQ6: fell mentally tense	3.09	0.967	−0.009	−0.381
MQ7: fell in a bad or low mood	3.11	0.952	−0.150	−0.304
MQ8: feel afraid without reason	3.64	1.042	−0.458	−0.373
MQ9: need repeated confirmation of completed tasks to feel secure	2.68	1.117	0.144	−0.765
MQ10: feel lonely even when being with others	3.49	1.107	−0.365	−0.557
MQ11: feel restless or uneasy	3.63	1.020	−0.429	−0.368
MQ12: feel empty, bored, or that the life has no meaning	3.66	1.125	−0.491	−0.547
MQ13: memory status	3.28	0.907	−0.192	−0.037
MQ14: be able to concentrate easily on one thing	3.46	0.876	−0.159	−0.194
MQ15: have the ability to think through or handle problems	3.55	0.782	−0.127	0.045
MQ16: self-rated overall mental health status	3.68	0.885	−0.262	−0.199

First, we carried out an analysis of internal consistency reliability and item-total correlations for the 15 items. An initial Cronbach’s alpha reliability coefficient (α) for the 15-item scale was 0.877 (Table 3). The item-total correlations for all ranged from 0.305 to 0.643. Within them, the coefficients of item 9 with total score correlation were quite low (r = 0.305), and deletion of the item did not reduce α value, suggesting comparative models for later structural analysis. After conducting EFA and CFA, a 3-factor model was confirmed with 14 items, and a second analysis of internal consistency was performed. The α reliability coefficients both in subscales and total scale were all regarded as an acceptable threshold over 0.7 [85,86]. Additionally, we calculated McDonald’s Omega (ω) for the M1, M2, M3 and the whole scale using the factor loadings and error variances, following the established formula for Omega calculation [87,88], and implemented using the SPSS macro developed by Hayes and Coutts [88]. All omega values exceeded 0.7, indicating acceptable internal consistency.

**Table 3:** Corrected item-total correlations and internal consistency reliability for scale (n = 3279).

Item	Corrected item-total correlation	Cronbach’s α if item delete	Cronbach’s α	ω	Dimensions
MQ1	0.643	0.865	0.897	0.898	M1: Positive emotion
MQ2	0.612	0.866			
MQ3	0.622	0.866			
MQ4	0.585	0.867			
MQ5	0.577	0.868			
MQ6	0.552	0.869	0.865 (0.876 if MQ9 deleted)	0.868 (0.878 if MQ9 deleted)	M2: Psychosocial symptoms and negative emotion
MQ7	0.611	0.866			
MQ8	0.578	0.867			
MQ9	0.305	**0.881**			
MQ10	0.562	0.868			
MQ11	0.627	0.865			
MQ12	0.598	0.866			
MQ13	0.334	0.878	0.746	0.750	M3: Cognitive function
MQ14	0.390	0.876			
MQ15	0.383	0.876			

Note. Total scale α = 0.877 (0.881 if MQ9 deleted); Total scale ω = 0.868 (0.873 if MQ9 deleted); ω was calculated using the formula: ω = (Σλᵢ)²/ [(Σλᵢ)² + Σθᵢ], where λᵢ is the standardized factor loading and θᵢ is the error variance (θᵢ = 1 – λᵢ²).

### 3.3. Exploratory factor analyses (Construct validity)

EFA was conducted initially using the 15-item mental health scale on the data (n = 1642) to identify the instrument’s dimensionality, and to assess the need for removal of some item(s) which may not contribute to the overall scale. The KMO and Bartlett’s test results (15 items, KMO = 0.903, p < 0.001) implied the data were suitable for factor analysis (See S3 Table in S1 File). The analysis confirmed the 3-dimensional model with previous studies [63], but initial factor loading was 0.576 and communality of item 9 was only 0.333(the acceptable index of an item should be over 0.6 and 0.4, respectively), suggesting that item 9 loaded independently of all other items [89]. Thus, EFA was subsequently conducted on 14 items, and still fit the 3-factor model, and the total explanation of 66.65% with the extraction of eigenvalues greater than 1.00, which indicated a good construct validity.

#### 3.3.1. Convergent validity.

Based on Pearson’s correlations, the inter-item correlations within each dimension were substantially higher than the correlations between items from different dimensions, indicating stronger associations among items within the same dimension. To examine convergence and validity in greater depth, we tested the value of average variances extracted (AVE) and composite reliability (CR). The minimum requirement for the AVE value is 0.5, and for the CR value is 0.7, and higher scores indicate higher convergent validity [90]. According to Table 4 (there are more details in S4 Table in S1 File), the test results of AVE values are all above 0.5 and the CR values are all above 0.7. Overall, this exhibited that each factor dimension possessed good convergent validity and composite reliability.

**Table 4:** Convergent validity and composite reliability test (14-item) (n = 1642).

			standardized factor loading	SE	P	CR	AVE
MQ1	<---	M1	0.845		<0.001	0.899	0.641
MQ2	<---	M1	0.844	0.024	<0.001		
MQ3	<---	M1	0.801	0.024	<0.001		
MQ4	<---	M1	0.710	0.025	<0.001		
MQ5	<---	M1	0.794	0.024	<0.001		
MQ6	<---	M2	0.652		<0.001	0.876	0.543
MQ7	<---	M2	0.702	0.044	<0.001		
MQ8	<---	M2	0.732	0.047	<0.001		
MQ10	<---	M2	0.726	0.051	<0.001		
MQ11	<---	M2	0.840	0.049	<0.001		
MQ12	<---	M2	0.754	0.052	<0.001		
MQ13	<---	M3	0.665		<0.001	0.765	0.521
MQ14	<---	M3	0.769	0.037	<0.001		
MQ15	<---	M3	0.727	0.032	<0.001		

#### 3.3.2. Discriminant validity.

Discriminant validity reflects the degree of differentiation between different variables. In accordance with Fornell and Larcker in their study [90], it was meaningful to compare the square root of the AVE value for different latent variables with the correlation coefficients between different latent variables. If the square root of the AVE for each latent variable is greater than the correlation coefficient between the latent variables (if the former value is higher), it demonstrates good discriminant validity. Table 5 shows the results. There was significant correlation among the three factors (latent variables M1, M2, & M3), but they also exhibited distinctions from one another.

**Table 5:** Discriminant validity (n = 1642).

factors	M1	M2	M3
M1	**0.801**		
M2	0.463**	**0.737**	
M3	0.541**	0.256**	**0.722**
AVE	0.641	0.543	0.521

Note. **p < 0.01; Diagonal: Square root of AVE.

### 3.4. Confirmatory factor analysis

#### 3.4.1. Three-factor Model Test.

**Fig 1:**
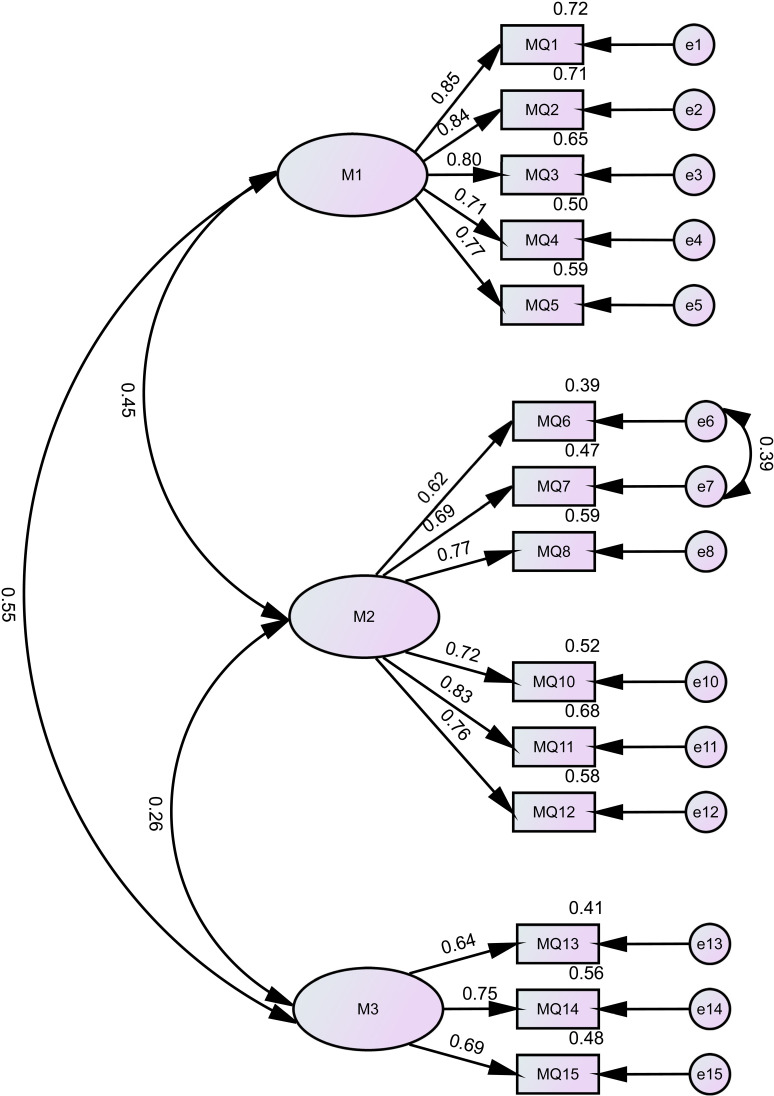
Diagram of confirmatory model: Regression coefficients for all significant paths at p < 0.001 level (n = 1637).

To evaluate the consistency of the estimated mental health status model with the data, we examined the goodness of fit by CFA with the other split-half data (n = 1637). Regarding complex data, combinational rules are recommended by researchers. A well-fitting model would be evidence of the value of CFI higher than 0.95, (around 0.9 is acceptable), RMSEA value close to 0.6 or less, and SRMR value close to 0.05 or less [91]. Whereas, values of RMSEA as high as 0.08 were also deemed a good fit model [92]. The CFA demonstrated support for the three-domain solution and the conceptual model of mental health status. The standard factor loadings concerning the three-factor model are displayed in Fig 1.

To ensure the adequacy of the sample size for the confirmatory factor analysis, a post hoc power analysis was conducted using Soper’s SEM sample size calculator [93]. The analysis, based on 14 observed variables and 3 latent factors, with a medium effect size (f² = 0.15), a power level of 0.95, and α = 0.05, indicated a minimum required sample size of 849. The CFA sample size (n = 1637) exceeded this requirement, demonstrating sufficient statistical power. The CFA results were specified for the 14 items, and model fit statistics are reported in Table 6. This primary model fit the data well, with fit indices of CFI = 0.96, TLI = 0.94, RMSEA = 0.07, SRMR = 0.04. According to the basis of modification indices, error variances are allowed with covariance between item 6 (feel emotionally tense) and item 7 (feel in low or bad mood). Hence, an adjustment was made, and then the model fit became even better than the primary fit, with fit indices of CFI = 0.97, TLI = 0.96, RMSEA = 0.06, SRMR = 0.03, leading to a well-fitting model.

**Table 6:** Model fit indices for the CFA analysis (n = 1637).

items		χ^2^/df	CFI	TLI	RMSEA	SRMR
		(around 5)	(≥ 0.95)	(≥ 0.95)	(≤ 0.06)	(≤ 0.05)
Primary 3 factor (n = 1637)		8.78	0.96	0.94	0.07	0.04
Adjusted model (n = 1637)		5.98	0.97	0.96	0.06	0.03
Gender	Male (n = 777)	3.28	0.97	0.96	0.05	0.04
	Female (n = 860)	3.71	0.97	0.96	0.06	0.03
Age	14-18 (n = 331)	2.70	0.96	0.94	0.07	0.04
	19-24 (n = 444)	1.96	0.98	0.97	0.05	0.04
	25-30 (n = 547)	2.93	0.96	0.95	0.06	0.05
	31-35 (n = 315)	2.49	0.95	0.94	0.07	0.05
Social Identity	students (n = 548)	2.96	0.96	0.96	0.06	0.04
	employed youth (n = 881)	3.89	0.96	0.96	0.06	0.04
	unemployed youth (n = 208)	1.95	0.95	0.94	0.07	0.05

#### 3.4.2. Factorial validation in subsamples.

CFA studies were separately conducted across the subsamples of age, gender, and youth social identity. The CFA results obtained (goodness of fit is shown in Table 6) were satisfactory for the subsamples, which suggested that the scale is usable and valid for different subgroups, with a good measurement invariance. Then, a multigroup CFA (MG-CFA) was conducted to examine a sequence of invariance across gender, age, and social identity groups and the results demonstrated robust support for metric and scalar invariance within the models (see S5 Table in S1 File). The unconstrained models for all groups showcased adequate model fits, indicating that the factor structures were appropriately specified for each group. The finding showed consistency in model parameters across diverse groups, which indicated good fit indices and minimal changes, fulfilling the criteria recommended to consider a model invariant with statistical indicators such as p-values less than 0.05 and minimal increments in TLI and NFI values below 0.05 further [94,95].

### 3.5. Concurrent validity

As mentioned earlier, the concurrent validity test was conducted by examining the correlations between mental health scale and the related psychological outcomes. The correlation coefficients between the three mental health subscales score, summed score of mental health scale, overall self-rated mental health, overall self-rated health status, sense of happiness and meaning in life were calculated and the results are presented in Table 7. All the correlation coefficients between variables were significant at the p < 0.001 level and varied from 0.230 to 0.825. Particularly, large positive correlations (r > 0.50) [84,96] were observed between mental health status (summed score) and SRMH, SRH, SH, respectively, and a moderate correlation with meaning in life. Additionally, moderate-to-large effect sizes (r > 0.3) were found between positive emotion with negative emotion, cognitive function, and all the other criteria. The coefficient results revealed good concurrent validity of mental health scale.

**Table 7:** Correlations between the Mental Health scale and its subscales with related psychological outcomes (n = 1637).

	M1	M2	M3	MH	SRMH	SRH	SH	ML
M1	1							
M2	.420^**^	1						
M3	.445^***^	.207^***^	1					
MH	.821^***^	.825^***^	.571^***^	1				
SRMH	.538^***^	.422^***^	.482^***^	.613^***^	1			
SRH	.533^***^	.328^***^	.366^***^	.528^***^	.506^***^	1		
SH	.599^***^	.366^***^	.260^***^	.556^***^	.387^***^	.387^***^	1	
ML	.470^***^	.230^***^	.430^***^	.459^***^	.379^***^	.381^***^	.357^***^	1

Note: M1 = positive emotion; M2 = psychosocial symptoms and negative emotion; M3 = cognitive function; MH = Mental health; SRMH = Overall self-rated mental health; SRH = Overall self-rated health status; SH = Sense of happiness; ML = Meaning in life; All values are Pearson’s correlation coefficients; *** p < 0.001 (two tailed).

## 4. Discussion

This study complements previous studies from three perspectives mainly. First, the study offers a comprehensive evaluation of the general mental health measurement among Chinese adolescents and young adults, revealing nuanced insights into the validity and applicability of this scale. The SRHMS was originally developed to evaluate health status encompassing physical and mental health for Chinese. This study, for the first time, specifically targets the mental health component within the scale, aiming to validate its effectiveness for a demographic that has been underrepresented in mental health research, particularly in China. To our knowledge, it is also used for the first time to systematically explore the psychometric properties and measurement invariance of a general mental health scale across representative youth groups in a Chinese environment. The analysis adopted the youth development data with a rigorous collecting process, comprising thousands of participants aged 14–35 years and reflecting a broad spectrum of the youth demographic, including students, employed youth, and unemployed youth. The measure is applicable to young people from various sociodemographic backgrounds, as it captures the general psychological symptoms including both positive emotion and negative aspects, along with the cognitive function occurrence during the period of youth development. The scale’s three-dimensional structure is confirmed. The multidimensional approach aligns with the global research’s advocacy for the understanding of mental health as not merely the absence of mental illness, but also the presence of positive psychological well-being. Confirming this structure emphasizes the complexity of mental health and the importance of assessment tools covering multiple related dimensions for a comprehensive understanding of individuals’ daily psychological states.

Second, the validated process contributes to the gaps highlighted in recent critiques. As Hodson referred, convergent validity and discriminant validity received short little attention in practical studies, despite their overtly recognized importance, and discriminant validity was particularly under-presented [97]. This study used several ways to thoroughly examine construct validity and shows strong levels of construct validity. The findings underscore its robust convergent and discriminant validity in assessing mental health among the Chinese youth population. Convergent validity was affirmed through the examination of AVE and CR for the scale’s three dimensions. The results show that all AVE values exceeded the 0.5 threshold, and CR values were above 0.7, indicating that each dimension possesses good convergent validity and reliability. Discriminant validity, which assesses the distinctiveness of different variables, was demonstrated by comparing the square root of the AVE for each latent variable against the correlation coefficients between latent variables. The analysis signifies good discriminant validity. This suggests that while the factors are correlated, they also maintain clear distinctions from one another, reinforcing the multidimensional nature of the mental health scale in capturing the complexities of mental health among the youth population in a culturally nuanced Chinese context. By demonstrating strong interrelations among indicators of theoretically similar constructs (evidence of convergent validity) and distinguishing between indicators of theoretically distinct constructs (evidence of discriminant validity), the study provides a clear framework for understanding the multifaceted nature of the constructs in general mental health that it aims to measure. This not only adheres to the traditional importance placed on both types of validity, as noted by Campbell and Fiske [98] and Lilienfeld and Strother [99], but also addresses the call by Spector for these validity types to be considered in relation to each other [100]. By providing robust evidence of both convergent and discriminant validity, this study fills a significant void in the body of literature, challenging the field’s reported shortfall in construct analysis, and ensures the reliability and applicability of the mental health scale through the necessity of such validation processes.

It should be noted that the item 9 (MQ9) “need repeated confirmation of completed tasks to feel secure” exhibits high variability with a standard deviation of 1.117 and a low factor loading below 0.4. This suggests that responses to this item are highly varied among participants, potentially indicating differing levels of dependency or anxiety traits across the sample. The low factor loading indicates a weak association with the underlying construct measured by the scale, suggesting that this item may not be effectively capturing the intended psychological trait or may be conceptually misaligned with the rest of the items in the scale. The result support the latest study for medical staff, but is inconsistent with the study of southern elderly population in China [57,58,63]. This item focuses on behavioral manifestation, specifically the need for repeated confirmation to feel secure, which may lead to a misalignment with other items on a psychological construct level. Additionally, within the cultural context of younger populations, this emphasis on behavioral manifestation can further contribute to its misfit with other elements of the psychological scale.

Third, the examination results of this study indicate good psychometric properties of the general mental health scale as a measure of youth mental status. The confirmation of the three-dimensional mental health scale addresses the cultural and contextual relevance of the measurement instrument, a crucial consideration given the variation in mental health perceptions and manifestations across different societies. Specifically, a principal component analysis (PCA) was carried out and 14 items with a 3-factor structure were identified. It accounts for 66.11% of the total variance in the scale. CFA analysis was then conducted and the results indicated satisfactory fit indices, and each item within the subscales accurately reflected its respective sub-construct, affirming the construct validity of the instrument. The following CFA result also supported the 14-item and 3-factor scale model. Additionally, with regards to reliability, good internal consistency is found both in full scale and subscales, with the values ranging from 0.746 to 0.897 and considered to be satisfactory.

Furthermore, concurrent validity and subgroup consistency across gender, age, and social identity were also conducted. Such validation is constantly lacking in literature, to the best of our knowledge. The CFA results showed a sufficient goodness of fit across three kinds of respective sub-groups, suggesting the stability of subgroup consistency. Along with the acceptable results of concurrent validity, that the mental health scale and its subscales were positively correlated with psychological health related outcomes, including overall self-rated mental health, overall self-rated health status, sense of happiness and meaning in life, the study further indicates that the mental health scale is a promising measurement instrument.

Overall, all the results demonstrate that the mental health scale shows adequate reliability and satisfactory validity. From the theoretical perspective, the study points to the value of the scale being utilized for extensive screening within community settings, and facilitates rapid self-assessment of mental health status among diverse youth groups. From a practical perspective, the development of the general mental health scale will benefit policy-making and targeted interventions to improve youth mental well-being in China. Nowadays, although there is high public awareness about the importance of youth mental health, and numerous relevant policies and strategies, there remains a significant gap in understanding the effectiveness of these initiatives and the actual changes in youth mental health status. In this sense, it is essential and critical to develop a reliable and valid measurement instrument that can be used to provide insights into the mental health status of young people. The 14-item SRMHS developed in this study can evaluate the general mental health status of the youth population, be applied across various contexts, and it is not time-consuming at all. It is warranted for policy-makers, practitioners, individuals, and any stakeholders to identify youth psychological status. The precise measurement tool identifies areas of concern, enabling more focused and effective support, and also ensures the ongoing monitoring and evaluation of targeted policies and programs over time, allocating resources more effectively, and adapting strategies to meet evolving needs.

Beyond the previously described contributions and significance, it is important to acknowledge certain limitations that warrant attention and future research. Data collection in this study was conducted through paper questionnaires, which were extensive in length due to the abundance of the youth development content covered. Consequently, the data collection led to omissions during the survey or errors in subsequent data input, thereby increasing the potential error for the sample. Therefore, data collection requires more strategies and support to improve the quality of questionnaire responses and data transcription. The 14-item SRMHS, which is originally derived from the SRHMS, while efficient and effective for quick assessments, might not encompass the full spectrum of mental health constructs or emerging issues within youth mental health. Expanding the scale, such as using the full version of the SRHMS, or developing complementary tools to cover a broader range of mental health understanding could be beneficial. Although the study attempts to account for subgroup consistency, there may still be nuances within subgroups (e.g., variations within the employed youth group based on educational levels) that were not fully explored. Future research could delve deeper into these distinctions to tailor more specific interventions. Furthermore, based on our discussions, it is evident that future research would benefit from the adoption of more sophisticated statistical analysis tools to enhance the rigor and credibility of data analysis. Applying advanced statistical methods such as multilevel modeling, structural equation modeling, or machine learning techniques could accurately assess and interpret complex data structures and relationships, thereby improving the reliability and applicability of the research findings.

Additionally, the study primarily offers a cross-sectional snapshot of mental health status among the youth. Longitudinal studies could provide deeper insights into the reliability and validity of the measurement instrument, and how mental health trajectories develop over time in response to interventions and changing social or personal circumstances. Such data are critical for developing and refining dynamic models of mental health that can inform ongoing policy and practice. In addition, while this study provides invaluable insights into the mental health of Chinese adolescents and young adults in a big city, the scale’s cultural and contextual specificity may limit its direct applicability to populations in different socio-cultural contexts. Future research could focus on cross-cultural validation studies and the scale’s implementation in different settings (e.g., rural areas, online platforms) and accessibility (including language adaptation, and literacy levels), to enhance the scale’s universality and applicability across diverse youth populations. Moreover, integrating the mental health measure into larger, multisectoral health and well-being studies could enhance our understanding of the interplay between mental health and other factors, such as physical health, educational achievements, and social relationships. This holistic approach is essential for developing comprehensive strategies to promote overall well-being among youth.

## 5. Conclusion

The validation of the general mental health scale in this study represents a significant step forward in the field of youth mental health research, particularly in the context of China. By providing a tool that is both scientifically robust and culturally sensitive, this research contributes to the global effort to understand and improve the mental health of Chinese young people.

This study’s emphasis on multidimensional assessment echoes global shifts towards more holistic approaches in mental health research and practice. Its development and validation of multidimensional assessment in a Chinese urban youth context provides a valuable model for similar endeavors worldwide, and encourages a broader, more inclusive approach to understanding mental health across different cultures and contexts. Future research should continue to explore and validate the dimensions of mental wellness across cultures, ensuring that interventions are both effective and culturally sensitive. Policymakers and practitioners must leverage these insights to design and implement mental health strategies that address the complex needs of adolescents, ultimately fostering a more resilient and mentally healthy youth population.

## Supporting information

S1 File Descriptive statistics and response distributions (S1 Table); Pearson’s correlation coefficients (S2 Table); Rotated factor loading scores from exploratory factor analysis (EFA; S3 Table); Convergent validity and composite reliability test for the 15-item scale (S4 Table); Model fit and measurement invariance of the mental health scale across gender, age, and social identity (S5 Table).
(XLS)
